# Modulation of monocyte matrix metalloproteinase-2 by breast adenocarcinoma cells

**DOI:** 10.1186/bcr1261

**Published:** 2005-06-14

**Authors:** Kristina A Szabo, Gurmit Singh

**Affiliations:** 1 Juravinski Cancer Centre, Hamilton, Ontario, Canada; 2 Department of Pathology and Molecular Medicine, McMaster University, Hamilton, Ontario, Canada

## Abstract

**Introduction:**

The presence of monocyte and macrophage cells in growing breast tumors, and the positive relationship between the degree of immune cell infiltration and tumor growth, suggest a possible paracrine growth regulatory function of immune cells in breast cancer.

**Method:**

To better understand the interaction between monocytes and breast cancer cells, *in vitro *matrix metalloproteinase and tissue inhibitor of metalloproteinase activity was assessed from the THP-1 myeloid cell line in response to conditioned media from two breast cancer cell lines, MCF-7 and MDA-MB-231.

**Results:**

Enzymography and immunoblotting revealed increased MMP-2 as well as increased levels of TIMP-1 and TIMP-2. Furthermore, a significant increase in the invasive potential of MCF-7 and MDA-MB-231 cells was noted in response to THP-1 cell-conditioned media.

**Conclusion:**

These data demonstrate that monocyte cells in the breast tumor microenvironment play an important role in the modulation of MMPs, which may have a significant effect on the control of tumor growth and metastatic spread.

## Introduction

There is a growing body of evidence to suggest that the tumor microenvironment is immunosuppressive [1,2]. This is perhaps as a result of selection for such an environment, which is a process recently termed immunoediting [3]. The growth of solid tumors has been likened to an aberration of the normal process of wound healing and, in consequence, immune cells may inadvertently aid tumor growth. The tumor microenvironment often contains a number of migratory haematopoietic cells that play pivotal roles in the progression and metastasis of tumors [4-8]. Monocyte and macrophage cells are prominent in the inflammatory infiltrate of tumors, often in considerable numbers [9-12], and it has been suggested that the presence of these cells may independently influence the metastatic potential of certain tumors [13].

The clinical significance of the mononuclear infiltrate that is often seen in breast cancer has remained the subject of continuous debate. In invasive breast carcinoma, the neoplastic cell population is often outnumbered by stromal cells such as tumor-associated macrophages (TAMs), which can comprise as much as 80% of the entire tumor-associated leukocyte (TAL) population [14], and more than 50% of the total tumor mass [15]. It appears that TAMs are actually required for the tumor to survive [16-19]. Other studies have also reported an overwhelming predominance of TAMs within the TAL population of both primary [12,20,21] and metastatic [22] breast carcinoma. Moreover, positive relationships between the presence of macrophages and lymph node metastasis [23], c-erbB2 [24], and increased expression of urokinase plasminogen activator (uPA) [25] in breast cancer have been reported.

The majority of studies that have attempted to correlate macrophage content with tumor severity have noted higher numbers of macrophages in conjunction with increasing tumor malignancy potential. Among the stromal cells, the presence of macrophage cells is frequently noted in aggressive malignant tumors, indicating a relation between macrophages and the degree of tumor cell differentiation [26-28]. These data are particularly compelling for breast, prostate, ovarian and cervical cancers. There is clinical data correlating a poor prognosis with the extent of macrophage infiltrate in breast cancer patients [26] as well as the differential cytotoxicity of macrophages from regressing and progressing tumors [29]. Monocytes represent precursor cells that serve as a source for the constant renewal of tissue macrophages on demand as well as in steady-state conditions. It is thought that monocytes in the peripheral circulation are recruited to the tumor site [30] by the release of chemotactic cytokines, and once recruited, the monocytes differentiate to become TAMs.

Matrix metalloproteinases (MMPs) comprise a family of zinc-containing endopeptidases that share structural domains and have the capacity to degrade extracellular matrix (ECM) components as well as to alter biological functions of ECM macromolecules [31]. The specific proteolytic targets of MMPs may include many other proteases, protease inhibitors, clotting factors, chemotactic molecules, latent growth factors, growth factor binding proteins, cell surface receptors, as well as cell-cell and cell-matrix adhesion molecules. Under physiological conditions, the activity of MMPs is tightly regulated to prevent excessive proteolytic activity and tissue destruction. Important sources of MMPs are immune cells, which utilize these enzymes to mediate extravasation into tissues during inflammation. Although initially it was assumed that the tumor cell was the origin of MMPs found in this environment, *in situ *hybridization techniques have shown that while some MMPs are expressed by tumor cells, MMPs are predominantly produced by adjacent host stromal and inflammatory cells in response to factors released by tumors [32,33]. The tumor cell MMPs may contribute to the invasive growth of the tumor while the stromal elements contribute to the remodelling process and the desmoplastic reaction that occurs in the tissue adjacent to the tumor [34].

MMPs produced by monocyte and macrophage cells, which have been implicated in tumor cell invasion and metastasis, may be enhanced by soluble factors from breast cancer cells. While tumor cells have been extensively studied, the roles of monocytes in the tumor microenvironment have not been well characterized. In an attempt to further understand the roles of monocytes in the tumor microenvironment, *in vitro *MMP and tissue inhibitor of metalloproteinase (TIMP) protein levels and enzymatic activity from THP-1 monocyte cells were assessed in response to the breast cancer cell lines MCF-7 and MDA-MB-231. An assessment of the invasive potential of these breast cancer cell lines in response to the monocyte cells was also conducted. Our results indicate that monocytes act as important regulators of ECM breakdown during tumor invasion and metastasis as a result of their ability to regulate MMP and TIMP production following their migration to the tumor site. Therefore, the basic mechanisms that regulate monocyte recruitment from the circulation into a tumor site and their production of MMPs and TIMPs are likely to be significant to breast cancer research.

## Materials and methods

### Cell lines and cell culture

The monocyte cell line THP-1, the estrogen receptor positive human breast cancer cell line MCF-7 and the estrogen receptor negative breast cancer cell line MDA-MB-231 were obtained from the American Type Culture Collection (Manassas, VA, USA). All cell lines were cultured under standard tissue culture conditions and tested negative for mycoplasma contamination. These cell lines were maintained in RPMI 1640 medium (Gibco, Grand Island, NY, USA), supplemented with 10% w/v fetal bovine serum (FBS) (Gibco), 1 mM sodium pyruvate, penicillin (100 units/ml) and streptomycin (100 units/ml) (Gibco) in a 5% CO_2 _incubator at 37°C. When experimental conditions called for the use of phenol red-free and serum-free media, the same RPMI medium was used as above but without the phenol red and FBS. The cells were only used a maximum of 15 passages and cells were grown to 80% confluence prior to experimentation. All media and reagents contained less than 0.06 endotoxin units/ml, confirmed by testing in our laboratory using the Limulus Amebocyte Lysate gel clot assay (Cambrex, East Rutherford, NJ, USA).

### Collection of conditioned media

Cultured cells were washed three times with phenol red-free, serum-free RPMI and incubated in this media for 48 h. Following incubation, the cells were harvested, the recovered cell number determined, and cell viability was assessed at the end of each culture period by Trypan Blue exclusion. Samples of tumor cell-derived conditioned media (CM) were collected and centrifuged to remove cell debris. Harvested CM was concentrated using the Amicon Ultra-4 centrifugal filter units with a nominal molecular weight limit of 10 kDa (Millipore, Bedford, MA, USA). The CM was immediately frozen at -80°C until enzymography or immunoblotting was performed.

### Treatment of THP-1 cells with conditioned media

For CM studies, THP-1 cells were grown to subconfluency (80% to 85%) in serum-supplemented media. The cells were then pelleted and washed three times in phenol red-free and serum-free RPMI before resuspension in this same media. Prior to experimentation, the concentration of the THP-1 cells was adjusted to 5 × 10^5 ^cells/ml by suspension in RPMI and the cells were seeded into six-well, flat bottom plates. The total volume of each well was 2.5 ml, with cells exposed to increasing volumes of MCF-7 and MDA-MB-231 breast cancer cell CM or incubated with control volumes of concentrated serum-free media for 48 h. After the 48 h incubation time, cells and debris were removed from the THP-1 supernatant by centrifugation. Following incubation, the recovered cell number was determined and cell viability established using Trypan Blue exclusion. Under the conditions in this study, the treatment of THP-1 cells with CM did not reduce the viability of the THP-1 cells.

### Western blotting

The CM protein levels were quantified using a protein assay (Bio-Rad, Hercules, CA, USA) and the results were compared with a standard curve of BSA concentrations. The protein concentrations were used to normalize the amount of CM loaded onto the gel; thus, the total protein loaded was equivalent in each lane. Loading buffer (5% w/v SDS, 0.225 M Tris-Cl pH 6.8, 50% w/v glycerol, 0.05% w/v bromophenol blue, 0.25 M dithiothreitol) was added to the CM and the mixture was denatured by boiling for 5 minutes. Samples were loaded onto a 10% SDS polyacrylamide gel with 10 μl of a wide-range colored protein molecular weight marker (Invitrogen, Carlsbad, CA, USA) also loaded onto the gel. The proteins were subsequently transferred from the gel onto a nitrocellulose membrane (Amersham Biosciences, Piscataway, NJ, USA). The membrane was incubated with mouse monoclonal anti-MMP-2 antibodies diluted 1:100 (Oncogene, Cambridge, MA, USA), and mouse monoclonal anti-TIMP-2, anti-TIMP-2 and anti-uPA antibodies diluted 1:400 (Oncogene). The membrane was subsequently incubated with mouse anti-rabbit IgG antibodies conjugated to horseradish peroxidase (HRP) or goat anti-mouse IgG antibodies HRP diluted 1:5000 (Santa Cruz Biotechnology, Santa Cruz, CA, USA). ECL chemiluminescent detection (Amersham Biosciences) was used to visualize the proteins.

### Enzymography

Aliquots of CM were mixed with sample buffer (0.5 M Tris-HCl pH 6.8, 10% w/v glycerol, 10% w/v SDS, and 0.1% w/v bromophenol blue) and were subjected to electrophoresis on a 10% SDS polyacrylamide gel containing 1 mg/ml gelatin (Sigma, St. Louis, MO, USA) or 1 mg/ml casein (Sigma). After electrophoresis, the gel was washed in 2.7% (v/v) Triton X-100 for 1 h at 37°C. The gels were then incubated in a developing buffer (50 mM Tris Base, 40 mM 6N HCl, 200 mM NaCl, 5 mM CaCl_2_·H_2_O, 0.02% v/v Brij 35) for 15 minutes at room temperature followed by an overnight incubation on a shaker at 37°C in the same buffer to allow digestion of the substrate. After digestion, the gels were rinsed briefly with deionized water and stained with 0.5% (w/v) Coomassie Brilliant Blue R-250 in 40% (v/v) ethanol and 10% (v/v) acetic acid for 1 h, followed by destaining in a mixture of 30% (v/v) ethanol and 10% (v/v) acetic acid. MMP proteolytic bands were identified by examining unstained regions on the substrate-stained background; intact protein substrate stained blue, leaving enzyme degraded bands transparent.

### Reverse enzymography

Protein standardized samples of CM were resolved in 10% SDS-PAGE containing 1 mg/ml gelatin (Sigma) and 1.5 μg/ml MMP-9 (Sigma). After gel electrophoresis, the gels were treated in the same manner as described for enzymography. For the reverse zymogram, the stained areas of the gel indicate enzymatic activity corresponding to the TIMP at that molecular weight.

### Motility and invasion assays

Cell motility was assessed using 24-well Matrigel Insert Chambers (Becton Dickinson Labware, Franklin Lakes, NJ, USA) with polycarbonate filters containing 8 μm pores coated with growth factor reduced matrigel matrix (50 μg/filter). MCF-7 and MDA-MB-231 cells were seeded at 5 × 10^4 ^cells/well in the upper compartment of each invasion chamber. The lower chambers contained serum-free media, serum-free media containing 10% v/v FBS, as well as THP-1 CM, 5 × 10^4 ^and 10 × 10^4 ^MCF-7, MDA-MB-231 and THP-1 cells per chamber. After 48 h, the top surface of the membrane was gently scrubbed with a cotton bud and cells on the undersurface were fixed and stained with the DiffQuick staining kit (Baxter, McGaw Park, Il, USA). The number of cells that had migrated to the undersurface of the filters was counted in five separate high-powered fields for each membrane. Values from the separate experiments containing all conditions were averaged and plotted on a bar graph ± their corresponding standard error.

### Statistical analysis of the data

All experiments were repeated a minimum of three times and representative results are shown. Student's t-test was used to compare results from the treated cells with the results from untreated control cells. Data are expressed as the mean ± standard error of the mean. Results were considered statistically significant at p < 0.05.

## Results

### Induction of MMP activity from THP-1 monocytes

**Figure 1:**
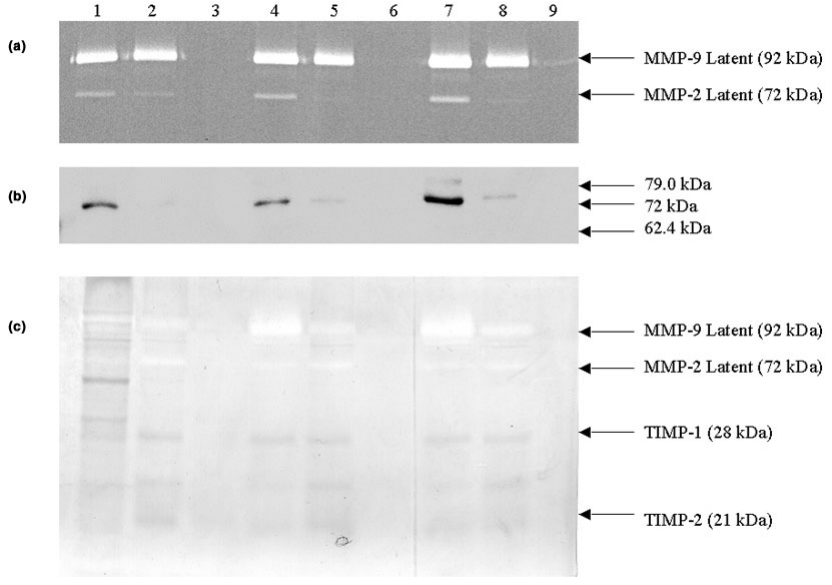
The effects of MCF-7 breast adenocarcinoma CM on MMP and TIMP activity from THP-1 monocytes. **(a) **Gelatin zymogram and **(b) **MMP-2 immunoblot showing the upregulation of monocyte MMP-2 after exposure to MCF-7 CM, and **(c) **a reverse zymogram to show that the activity of monocyte TIMP-1 and -2 remained the same upon treatment. Lanes 1, 4, and 7 contain CM from THP-1 cells in serum-free media grown in the presence of 100 μl, 200 μl and 300 μl concentrated MCF-7 CM, respectively. Lanes 2, 5, and 8 contain CM from THP-1 cells in serum-free media grown in the presence of 100 μl, 200 μl and 300 μl concentrated serum-free media, respectively. Lanes 3, 6, and 9 contain serum-free media with 100 μl, 200 μl and 300 μl, respectively, of MCF-7 CM in the absence of THP-1 cells. These figures are representative of three independent experiments carried out in duplicate, each of which demonstrates similar results.

**Figure 2:**
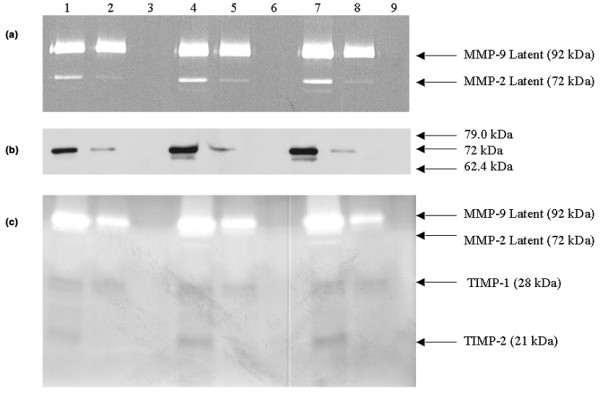
The effects of MDA-MB-231 breast adenocarcinoma CM on MMP and TIMP activity from THP-1 monocytes. **(a) **Gelatin zymogram and **(b) **MMP-2 immunoblot showing the upregulation of monocyte MMP-2 after exposure to MDA-MB-231 CM, and **(c) **a reverse zymogram to show that the activity of monocyte TIMP-2 was also upregulated upon exposure to MDA-MB-231 CM, although TIMP-1 activity remained the same upon treatment. Lanes 1, 4, and 7 contain CM from THP-1 cells in serum-free media grown in the presence of 100 μl, 200 μl and 300 μl concentrated MDA-MB-231 CM, respectively. Lanes 2, 5, and 8 contain CM from THP-1 cells in serum-free media grown in the presence of 100 μl, 200 μl and 300 μl concentrated serum-free media, respectively. Lanes 3, 6, and 9 contain serum-free media with 100 μl, 200 μl and 300 μl, respectively, of MDA-MB-231 CM in the absence of THP-1 cells. These figures are representative of three independent experiments carried out in duplicate, each of which demonstrates similar results.

MCF-7 (Fig. 1a) and MDA-MB-231 (Fig. 2a) CM elicited enhanced MMP-2 activity by THP-1 monocytes, as measured by gelatin zymography, when compared to either unstimulated controls or to the levels of the enzyme in the CM alone. Distinct gelatinolytic bands were observed at calculated M_r _72,000, and 92,000, corresponding to the latent 72 kDa type IV collagenase (MMP-2), and the latent form of 92 kDa type IV collagenase (MMP-9), respectively. At the highest concentration of CM (300 μl) the enzymatic effect was the greatest. This coincided with an increase in expression of MMP-2 (Figs 1b and 2b) protein levels produced by the monocyte cells as determined by immunoblot analysis. In addition, the presence of casein-degrading MMPs was analysed by enzymography, although there were no appreciable levels of MMP-1 or MMP-7 produced. Western blot analysis confirmed this absence of MMP-7 and, furthermore, the basal level MMP-9 and uPA produced by the monocyte cells was not changed upon exposure to the breast cancer CM (results not shown).

### Induction of TIMP activity from THP-1 monocytes

**Figure 3:**
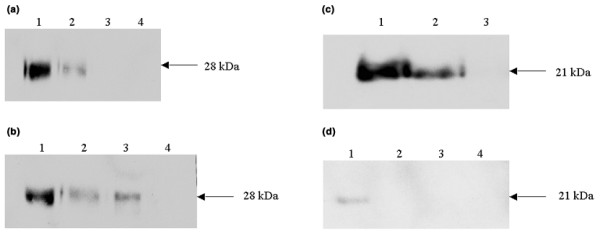
MCF-7 and MDA-MB-231 upregulation of TIMP-1 and TIMP-2 protein levels from THP-1 monocytes. Immunoblots representative of three independent experiments carried out in duplicate using monoclonal **(a,b) **TIMP-1 and **(c,d) **TIMP-2 antibodies to show an increased production of both proteins by monocyte cells in response to MCF-7 and MDA-MB-231 CM. Lane 1 contains CM from THP-1 cells in serum-free media grown in the presence of 300 μl concentrated **(a,c) **MCF-7 CM and **(b,d) **MDA-MB-231 CM. Lane 2 contains CM from THP-1 cells in serum-free media grown in the presence of 300 μl concentrated serum-free media. Lane 3 contains serum-free media with 300 μl of **(a,c) **MCF-7 CM and **(b,d) **MDA-MB-231 CM in the absence of THP-1 cells. Lane 4 contains unconcentrated **(a) **MCF-7 and **(b,d) **MDA-MB-231 CM.

In addition to the contribution by MMPs, the degree of connective tissue destruction is also influenced by TIMPs. Reverse enzymography using MMP-9 as the degrading source revealed TIMP-1 and TIMP-2 activity (Figs 1c and 2c) at M_r _28,000 and 21,000, respectively. There was no difference in the level of monocyte TIMP-1 or TIMP-2 inhibition between the control and MCF-7 CM treated cells. Upon exposure to the MDA-MB-231 CM, however, there was an increased level of TIMP-2 inhibition (Fig. 2c). In order to compare the inhibitory activity of TIMP-1 and TIMP-2 with the protein levels, immunoblot analysis was performed on the samples at the 300 μl optimal concentration. The THP-1 monocytes produced enhanced TIMP-1 (Fig. 3a,b) and TIMP-2 (Fig. 3c,d) protein levels; thus a small increase in TIMP-1 expression as well as a more substantial increase in TIMP-2 was observed.

### Motility and invasive potential of MCF-7 and MDA-MB-231 cells in response to monocytes

**Figure 4:**
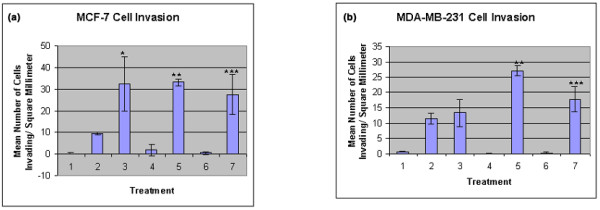
The effects of monocyte cells and CM on the invasive potential of breast adenocarcinoma cells. Graphs showing an increase in the number of **(a) **MCF-7 and **(b) **MDA-MB-231 cells that migrated through matrigel-coated membranes in response to monocyte cell CM. The following conditions were used in the lower chamber: 1, serum-free media; 2, 10% FBS in serum-free media; 3, THP-1 cell-conditioned media; 4, **(a) **5 × 10^4 ^MCF-7 cells, **(b) **5 × 10^4 ^MDA-MB-231 cells; 5, 5 × 10^4 ^THP-1 cells; 6, **(a) **10 × 10^4 ^MCF-7 cells, **(b) **10 × 10^4 ^MDA-MB-231 cells; 7, 10 × 10^4 ^THP-1 cells. *p < 0.05 compared to condition 2; **p < 0.05 compared to condition 4; ***p < 0.05 compared to condition 6. The values are the means of three experiments and the error bars are the standard error of the mean.

The previous results, and our interest in the ability of monocytes to degrade the ECM, raised the question of whether or not monocytes increase the invasive potential of breast cancer cells. The presence of THP-1 CM led to a significant increase (p < 0.05) in MCF-7 (Fig. 4a) cell invasion in comparison to the serum-free media control. The presence of 5 × 10^4 ^THP-1 cells in the lower chamber also led to a significant increase (p < 0.05) in the number of MCF-7 (Fig. 4a) and MDA-MB-231 (Fig. 4b) cells that invaded through the matrigel membrane. The invasive potential of the breast cancer cells was maintained, however, as the concentration of the monocyte cells was doubled.

## Discussion

The expression of components of the matrix degrading protease system by tumor stromal cells indicates that the tumor stroma does not merely play a passive role in cancer progression. Rather, it may in fact actively participate in the process of cancer invasion. We propose that it is the mixed population of cancer cells and recruited stromal cells that produce the matrix degrading protease components in order to facilitate the destruction of the surrounding normal tissue, which allows for malignant cell invasion. Our present data may partially explain why MMPs are often predominantly expressed by the stromal cells that surround invasive neoplastic cells. Specifically, monocytes may play an important role in tumor invasion and metastasis through their MMP proteolytic activity.

MMPs are usually expressed and secreted by cells as inactive enzymes, and further proteolytic processing is necessary to convert them into their active forms. The degradation of the ECM, especially basement membrane type IV collagen, is considered a key event for tumor cell invasion and metastasis. Specifically in colorectal cancer, MMP-9 is derived principally from stromal monocytes [35,36] and a high level of MMP-9 in tumor versus paired normal mucosa is an independent predictor of poor prognosis [37]. Moreover, there is a connection between the expression of TIMP-2 and MMP-2 under many physiological and pathological conditions, suggesting that TIMP-2 regulates MMP-2 activity [38,39]. Furthermore, TIMP-1 and TIMP-2 over-expression has been associated with malignant breast tumor behaviour *in vivo *[40,41]. Immunohistochemical studies have indicated that MMP-2 is highly expressed in more invasive and metastatic cancer tissues [42]. The fact that MMP-2 is often associated with adjacent normal tissues rather than the tumor cells themselves suggests that neoplastic cells can use MMPs produced by normal cells to facilitate their egress from the tumor mass and potentially their entry into new sites [43,44]. Thus, the breast cancer microenvironment may affect the monocyte MMP/TIMP balance and, consequently, play a role in the ECM breakdown.

Considering the high concentration of infiltrating immune cells in the breast cancer microenvironment, we examined whether breast cancer cells modulate the reactivity of monocyte cells by altering their production of MMPs and TIMPs. The data presented here suggest that monocyte-derived MMP, notably MMP-2, may play an important role in invasive processes. Both the protein levels and enzymatic activity of MMP-2 were elevated in response to concentrated MCF-7 and MDA-MB-231 CM. TIMP-1 and TIMP-2 protein levels were both increased in the CM treated cells. A study examining the effect of MCF-7 cells on human dermal fibroblasts found a similar observation, in that the breast cancer cells augmented the production of proMMP-1, -2, and -3 as well as TIMP-1 by fibroblast cells [45]. In addition, others have shown that contact between MDA-MB-231 cells and bone marrow derived fibroblasts resulted in an increase in the concentration of MMP-2 in the culture supernatant [46]. These findings are consistent with the observation that fibroblast cells promote tumor progression in animal models through their production of MMPs [47-49] and the findings emphasize the importance of tumor-host interactions during cancer progression. A recent study by Blot and colleagues [50] provides additional evidence on the role of monocytes in breast cancer. The authors cultured peripheral monocytes with MDA-MB-231 breast cancer cells and noticed an increase in MMP-9 expression [50]. Thus, the ability of MCF-7 and MDA-MB-231 cells to augment the production of MMPs in surrounding normal cells is likely one of the important properties for cell invasion and metastasis by these two breast cancer cell lines. However, since the THP-1 monocyte cell line differs in many respects from TAMs, further study is needed to assess the *in vivo *activities of monocyte cells and to determine if MMP-2 is upregulated by monocytes in the breast cancer microenvironment *in vivo*.

Collectively, these findings shed new light on the role of tumor-associated monocytes in the regulation of breast tumor development, invasion and metastasis. Increased MMP production has been shown to break down the basement membrane around pre-invasive tumors, thereby enhancing the ability of tumor cells to escape into the surrounding stroma. Thus, it is becoming apparent that stromal cells in the tumor microenvironment play an important role in allowing the tumor to express its full neoplastic phenotype. Consequently, further studies could reveal many new pathophysiological implications of MMPs in their regulation of immune cells, cytokine and chemokine networks, and matrix proteolysis during the metastatic process.

## Conclusion

This study supports the hypothesis that tumor cell-host stromal cell interactions play a critical role in the proteolytic cascade required for tumor progression. Here we have focussed on one type of solid tumor, carcinoma of the breast. Monocyte MMP-2 enzymatic activity and MMP-2 as well as TIMP-1 and TIMP-2 protein levels were increased in response to soluble factors from MCF-7 and MDA-MB-231 breast cancer cells. Furthermore, the breast cancer cells displayed significantly enhanced *in vitro *invasion in response to monocyte CM. These data provide evidence for a potentially important function of monocytes in the modulation of MMPs and degradation of connective tissue in neoplastic disease.

## Abbreviations

BSA = bovine serum albumin; CM = conditioned media; ECM = extracellular matrix; FBS = fetal bovine serum; HRP = horse radish peroxidase; MMP = matrix metalloproteinase; TAL = tumor-associated leukocyte; TAM = tumor-associated macrophage; TIMP = tissue-inhibitor of matrix metalloproteinase; uPA = urokinase plasminogen activator.

## Competing interests

The authors declare that they have no competing interests.

## Authors' contributions

KS conceived and designed the study, performed analysis, interpreted the data and drafted the article. GS coordinated the study and contributed to the design of the study, also taking a role in supervising and final approval of the manuscript.
